# Supplementary material for: Phase-transition tailored nanoporous zinc metal electrodes for rechargeable alkaline zinc-nickel oxide hydroxide and zinc-air batteries
Source: Nat Commun. 2022 May 24;13:2870. doi: 10.1038/s41467-022-30616-w (PMC9130287; doi:10.1038/s41467-022-30616-w)
Supplement: Supplementary file 1 — Supplementary information [file 41467_2022_30616_MOESM1_ESM.pdf]

Supplementary Information

Phase-transition tailored nanoporous zinc metal electrodes for rechargeable alkaline zinc-nickel oxide hydroxide and zinc-air batteries

Liangyu Li[1,2], Yung Chak Anson Tsang[3], Diwen Xiao[1], Guoyin Zhu[4], Chunyi Zhi[5], Qing Chen[1,2,6]*

[1]Department of Mechanical and Aerospace Engineering, the Hong Kong University of Science and Technology, Clear Water Bay, Kowloon, Hong Kong.

[2]The Energy Institute, the Hong Kong University of Science and Technology, Clear Water Bay, Kowloon, Hong Kong.

[3]George W. Woodruff School of Mechanical Engineering, Georgia Institute of Technology, Atlanta, GA, USA.

[4]School of Chemistry and Materials Science, Institute of Advanced Materials and Flexible Electronics (IAMFE), Nanjing University of Information Science and Technology, Nanjing, China.

[5]Department of Materials Science and Engineering, City University of Hong Kong, Kowloon, Hong Kong.

[6]Department of Chemistry, the Hong Kong University of Science and Technology, Clear Water Bay, Kowloon, Hong Kong.

*Correspondence addressed to chenqing@ust.hk.

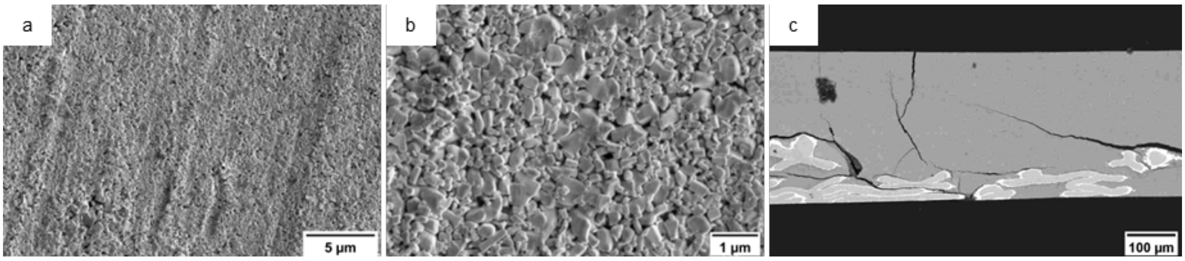

Supplementary Figure 1. (a, b) Surface SEM images of the pristine electrode compacted by ZnO particles under different magnifications. (c) Cross-section image with the epoxy resin filling, where the bright parts are the Sn plated Cu foam substrate, the gray parts are the compact ZnO powders.

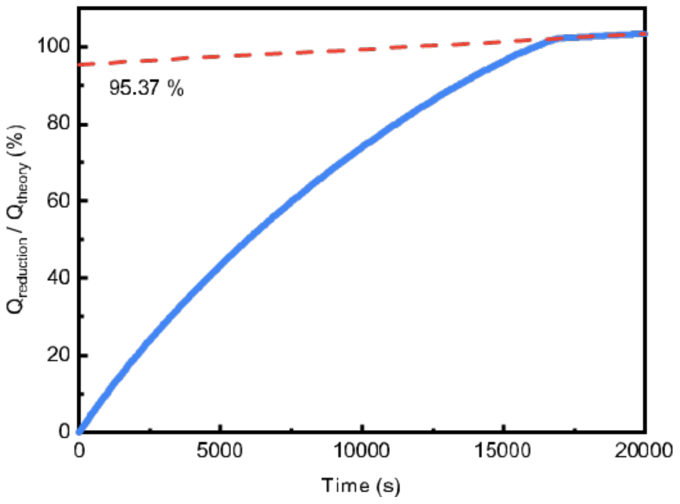

Supplementary Figure 2. The normalized chronocoulometric curve for the ZnO to Zn transition. The percentage of ZnO reduced was calculated based on the ratio between the charge of reduction ($Q_{reduction}$) and the theoretical charge if all the ZnO was reduced (Q_{theory}, calculated based on the amount of ZnO and Faraday's law). $Q_{reduction}$ was estimated by subtracting the total charge at the postulated point of completion (the point of an abrupt slope change) with the cut-off for hydrogen evolution. The cut-off for hydrogen evolution was estimated by extrapolating the chronocoulometric curve after the completion point to the beginning of the experiment, assuming constant current of hydrogen evolution.

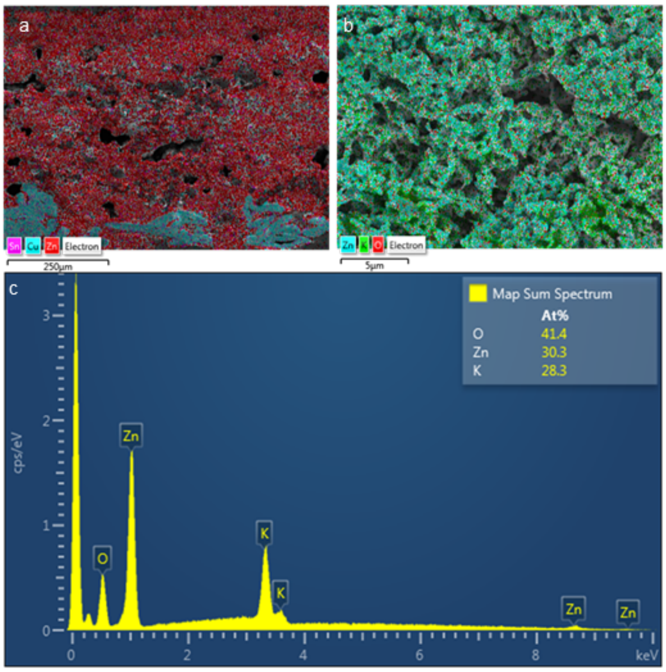

Supplementary Figure 3. Morphological and chemical characterizations of the NP Zn. (a) EDS of the cross-section of the NP Zn filled with the epoxy with the distribution of Zn, Cu and Sn colorized according to the bottom left. (b) EDS of the surface of the NP Zn and (c) the corresponding quantitative compositional analysis of the area in b. Excluding carbon, Zn, K, and O were identified as the main components, the latter two of which were likely from the residue of electrolyte difficult to completely remove especially from the pores. No Cu signal can be detected.

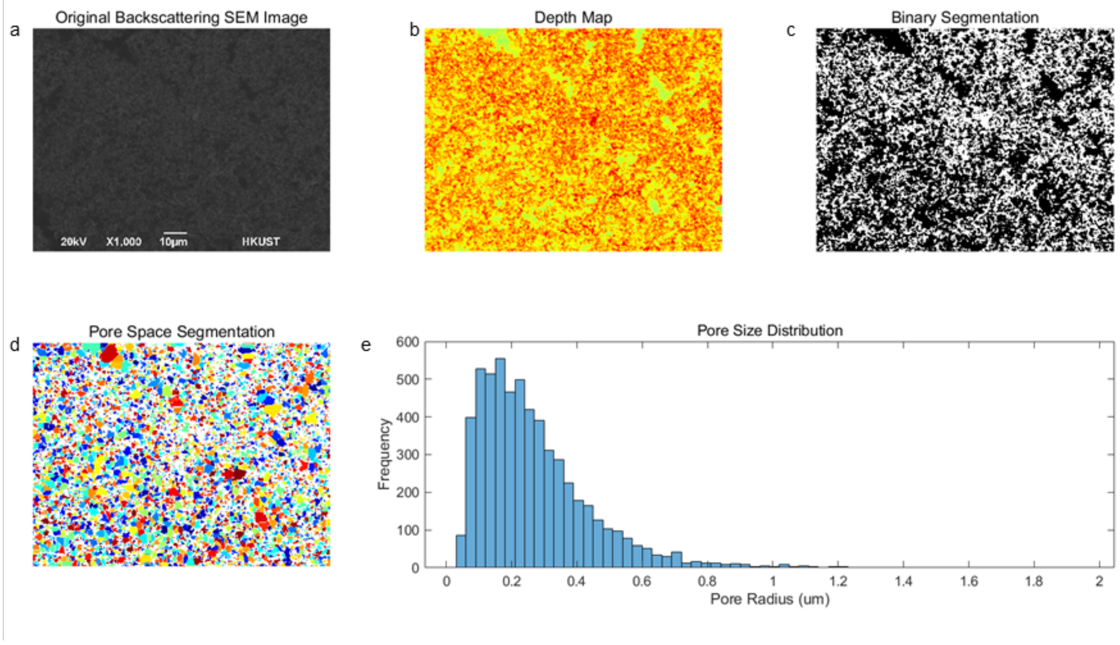

Supplementary Figure 4. The process of analysis for the porosity and pore size distribution of the NP Zn. Using a MATLAB package (SEM Image Porosity and Pore Size), the darkest portion in (a) representing the pore space of the SEM image can be detected with multi-level thresholding (shown as depth map in b). Then the image was transformed to a binarized version (c). The fraction of the black pixels in the binarized image is taken as the porosity, assuming the structure to be isotropic. The pore space is segmented (colorized with the Watershed algorithm as shown in d). We then performed the pore counting and size measurement to attain the size distribution as shown in (e).

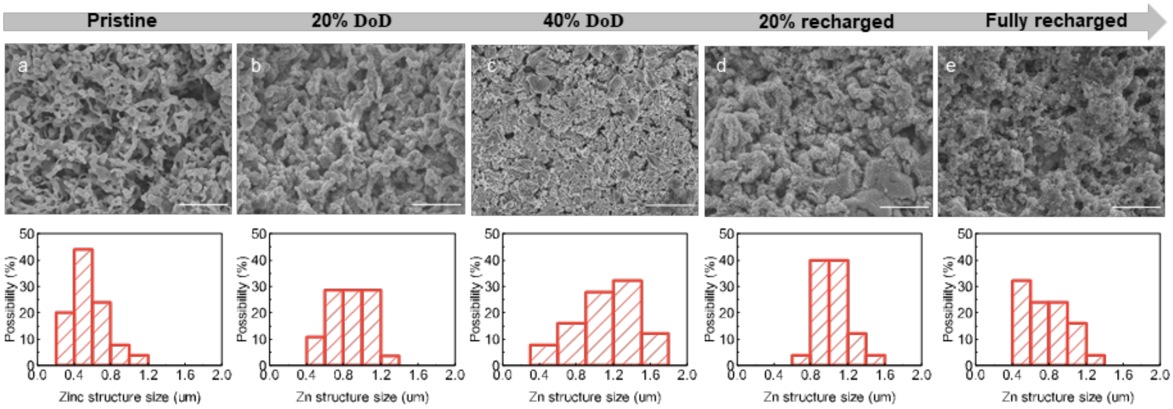

Supplementary Figure 5. SEM images of the NP Zn anode at the same stages (as shown in the arrow) in the corresponding images in Figure 3d, but at lower magnifications to show the uniformity. Below each image is a statistical analysis of the zinc structure size based on 25 randomly chosen areas from the image. The mean value increases in charging and decreases back in discharging, while the distribution is relatively stable. The scale bars are 5 µm.

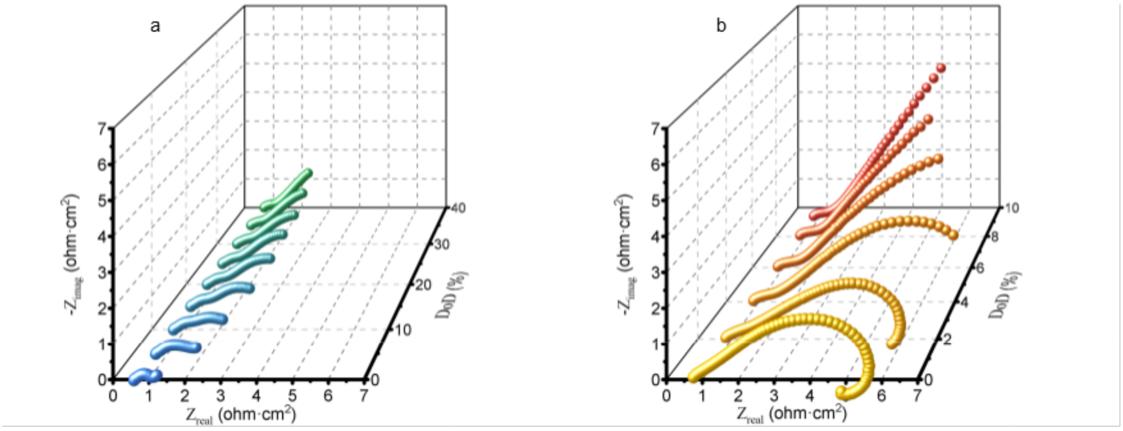

Supplementary Figure 6. EIS of the full cell with (a) the NP Zn anode and (b) the Zn powder anode, attained under the same condition as in Figure 4b and c.

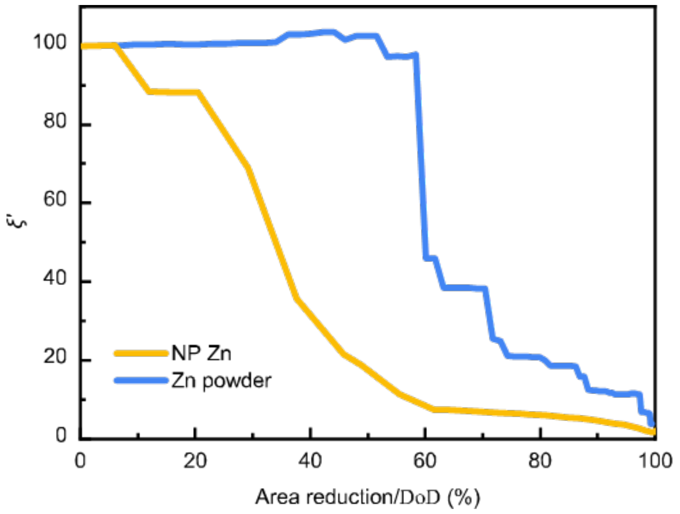

Supplementary Figure 7. Correlation length ξ' of NP Zn and Zn powder. Images of the structures at different DoD's were first generated using the same erosion algorithm as in Figure 4d – g. A MATLAB package (Fractal analysis) was used for estimating of the gyration radius (R_s) of every structural component (connected black pixels of the Zn phase) in the images via

$$R_s^2 = \langle |r_i - r_{cm}|^2 \rangle = \frac{1}{s}\sum_{i=1}^{s} |r_i - r_{cm}|^2$$

where s is the number of pixels in the component, i an integer, and $r_{cm} = \frac{1}{s}\sum_{i=1}^{s} r_i$ defines the position of the mass center of the component. The correlation length ξ' is then calculated as the average distance between two pixels within a component

$$\xi^2 = \frac{\sum_s s^2 n_s R_s^2}{\sum_s s^2 n_s}$$

where n_s is the number of components with the size s. The larger ξ', the more widely spanned the clusters, and the higher continuity in the solid phase.

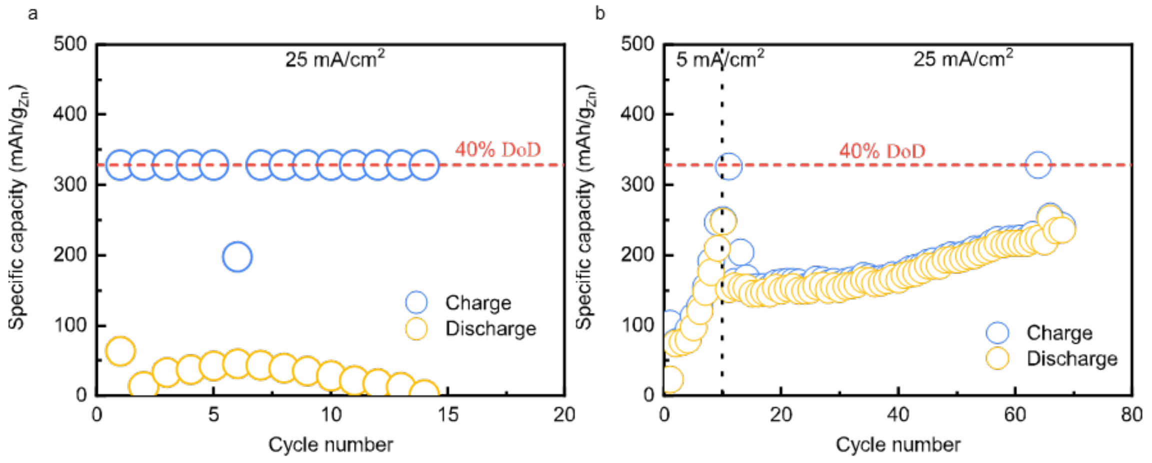

Supplementary Figure 8. The cycle performance of Zn||NiOOH battery with an anode comprising 85% Zn powder and 10% ZnO particles. (a) Charge and discharge specific capacity of the anode vs. the cycle number, attained under the same condition as in Figure 5c. The anode failed to deliver the designated 40% DoD (red dashed line), likely because the electrolyte (6 M KOH) did not suit ZnO and the poorly conductive ZnO prevented the utilization of Zn. (b) The performance attained by adding 10 cycles at 5 mA/cm^2 at the beginning (those before the black dashed line), as the common practice to activate ZnO powder anodes. The performance was better compared to (a), but still worse than that of the NP Zn or Zn powder anode, likely because of the non-uniform structural evolution in the activation process. We thus chose the Zn powder anode as the control group.

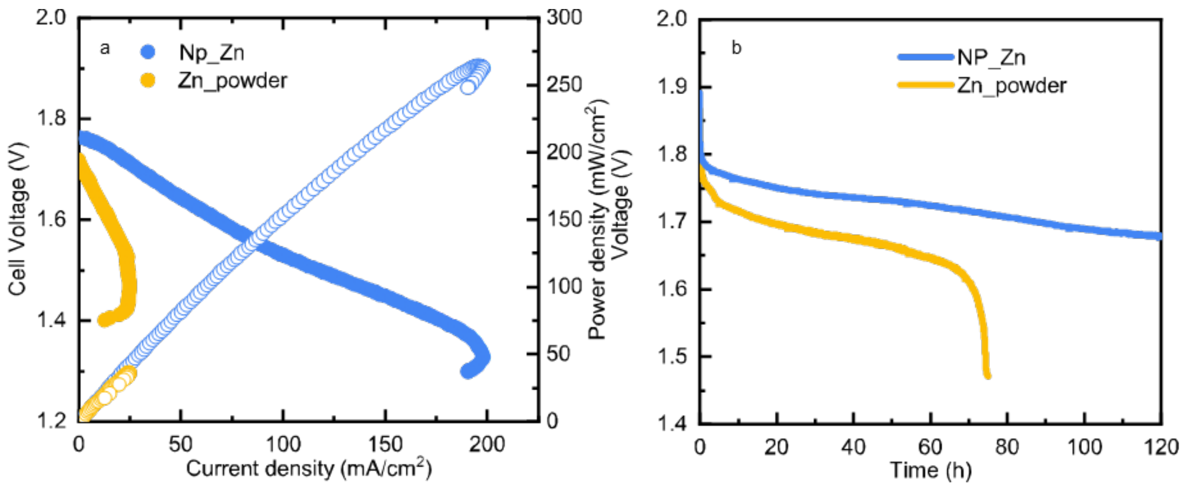

Supplementary Figure 9. (a) Polarization curves of the two anodes in Zn||NiOOH batteries. (b) The stability of the open-circuit voltage of Zn||NiOOH batteries over time.

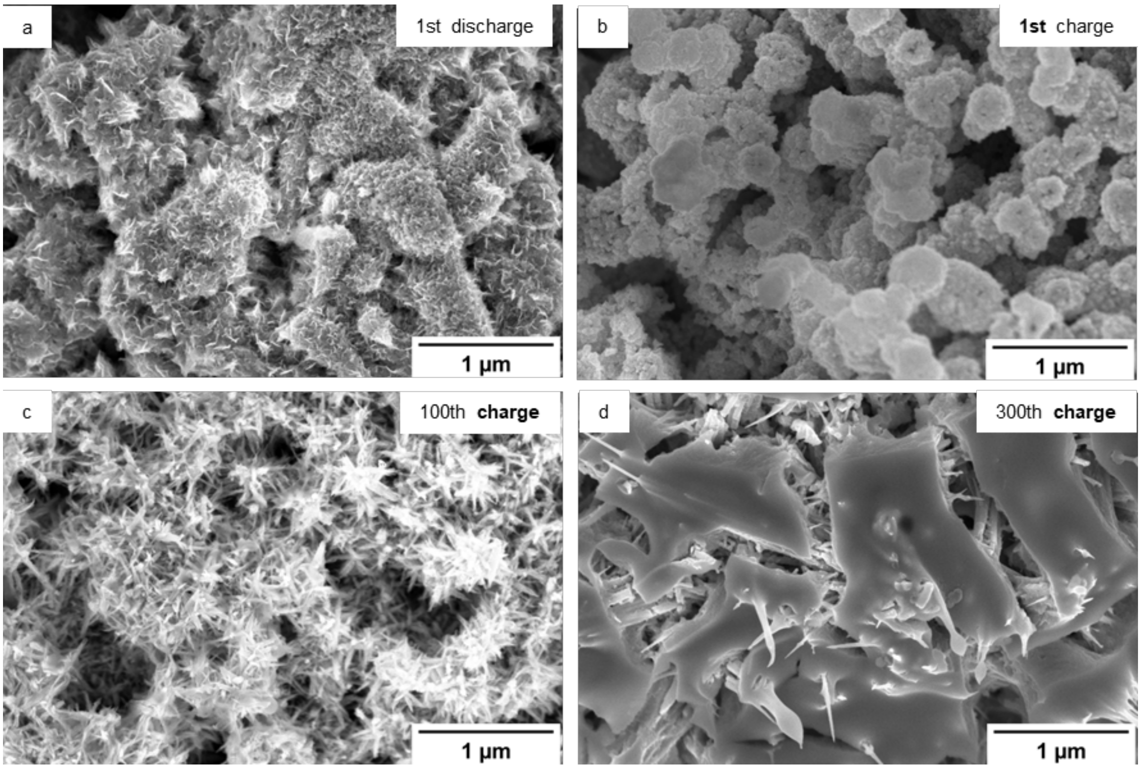

Supplementary Figure 10. Ex situ postmortem SEM micrographs of the nanoporous zinc metal electrode sampled at various cell cycles. The cell was cycled at a current density of 25 mA/cm^2 for both charge and discharge. The SEM micrograph in Supplementary Figure 10a was captured at fully discharged state. SEM micrographs in Supplementary Figure 10b-10d were captured at fully charged state of 1st, 100th and 300th cycles.

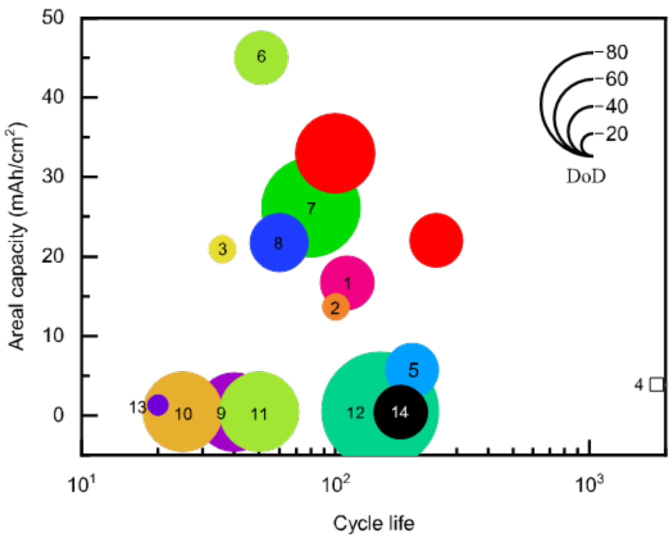

Supplementary Figure 11. A comparison of the Zn||NiOOH battery performance of this work (in red solid circles) with previous reports (numbered as in the Supplementary References) in terms of the cycle life vs. the areal capacity, with the values of DoD indicated as the sizes of the points. The cycle life corresponds to either the point of 80% capacity retention or the longest cycle reported in the work. Details can be found in Supplementary Table 1.

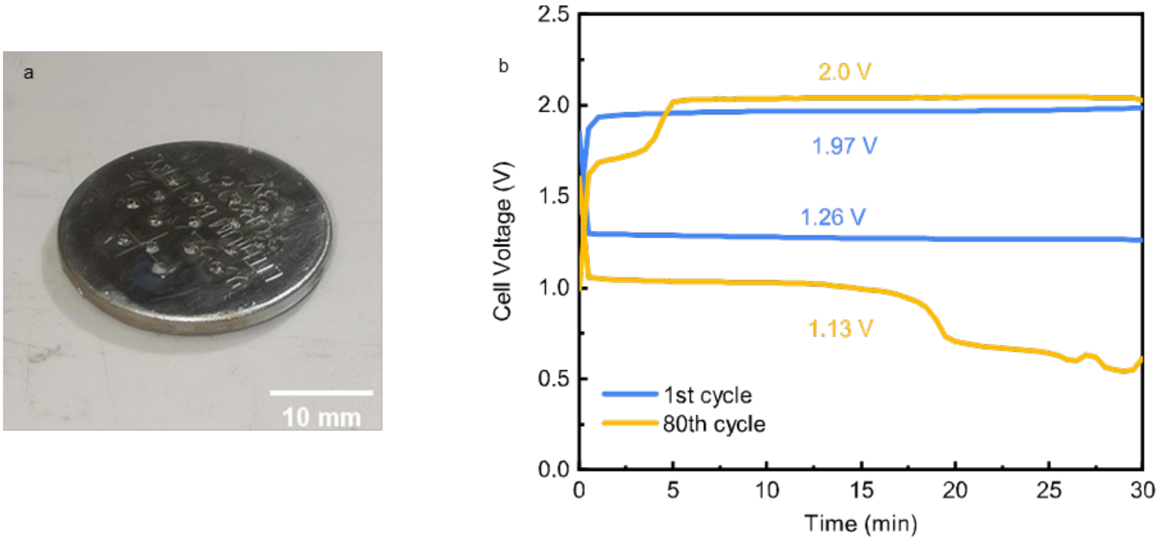

Supplementary Figure 12. (a) Photographic picture of the Zn-air battery. (b) Charging and discharging voltages vs. time for the Zn-air battery with the NP Zn anode in the 1st and 80th cycles. Since the charging and discharging capacities were set the same, the energy efficiency is proportional to the ratio between the areas under discharging and charging curves.

Supplementary table 1. The comparison of Zn||NiOOH battery performance.

Cell Type	DoD (%)	Areal Capacity (mAh/cm^2)[a]	Cycle life[b]	Supplementary Ref. No.
Nanoporous Zn<br>Zn||NiOOH<br>(alkaline aqueous coin cell)	40<br>60	20<br>30	250[b1]<br>100[b1]	This work
Zn sponge<br>Zn||NiOOH<br>(alkaline aqueous coin cell)	40	16.7	111[b2]	1
Zn sponge<br>Zn||Ag_2O<br>(alkaline aqueous coin cell)	20	13.6	100[b2]	2
Zn sponge<br>Zn||Ag_2O<br>(alkaline aqueous coin cell)	20	20.9	36[b2]	3
Zn/ZnO(85/10)<br>Zn/ZnO(85/10)||MnO_2<br>(alkaline aqueous home-made cell)	1.4	3.8	2000[b2]	4
Nanoporous Zn<br>Zn||NiOOH	40	5.7	200[b2]	5

(alkaline aqueous coin cell)				
Zn sponge<br>Zn‖NiOOH<br>(alkaline aqueous coin cell)	40	45	51[b2]	6
ZnO microsphere<br>ZnO‖$Ni(OH)_2$<br>(alkaline aqueous home-made cell)	74	26.19	80[b1]	7
ZnO@ppy<br>ZnO@ppy‖Co-Fe/C based air cathode<br>(alkaline aqueous stack Zn-air cell)	44	21.75	60[b2]	8
ZnO@C<br>ZnO@C‖$Ni(OH)_2$<br>(alkaline aqueous coin cell)	59	0.45	40[b2]	9
ZnO@TiN_xO_y<br>ZnO@TiN_xO_y‖$Ni(OH)_2$<br>(alkaline aqueous coin cell)	60	0.44	25[b1]	10
Zn-pome<br>Zn-pome‖$Ni(OH)_2$<br>(alkaline aqueous coin cell)	60	0.45	50[b2]	11
ZnO lasagna<br>ZnO lasagna‖$Ni(OH)_2$<br>(alkaline aqueous coin cell)	88	0.58	150[b2]	12

Zn@GO<br>Zn@GO‖NiOOH<br>(alkaline aqueous coin cell)	15.4	1.27	20[b2]	13
ZnO@TiO_2<br>ZnO@TiO_2‖NiOOH/$Ni(OH)_2$<br>(alkaline aqueous coin cell)	40	0.4	180[b2]	14

a: The area capacity is calculated with the first discharging step.

b: The cycle life corresponds to (b1) the point of 80% capacity retention or (b2) the longest cycle reported in the literature.

Supplementary References:

1. Parker, J. F. *et al.* Rechargeable nickel–3D zinc batteries: An energy-dense, safer alternative to lithium-ion. *Science* **356**, 415–418 (2017).

2. Ko, J. S. *et al.* Robust 3D Zn Sponges Enable High-Power, Energy-Dense Alkaline Batteries. *ACS Appl. Energy Mater.* **2**, 212–216 (2019).

3. Parker, J. F., Chervin, C. N., Nelson, E. S., Rolison, D. R. & Long, J. W. Wiring zinc in three dimensions re-writes battery performance—dendrite-free cycling. *Energy Env. Sci* **7**, 1117–1124 (2014).

4. Turney, D. E. *et al.* Rechargeable Zinc Alkaline Anodes for Long-Cycle Energy Storage. *Chem. Mater.* **29**, 4819–4832 (2017).

5. Wang, C., Zhu, G., Liu, P. & Chen, Q. Monolithic Nanoporous Zn Anode for Rechargeable Alkaline Batteries. *ACS Nano* **14**, 2404–2411 (2020).

6. Hopkins, B. J. *et al.* Fabricating architected zinc electrodes with unprecedented volumetric capacity in rechargeable alkaline cells. *Energy Storage Mater.* **27**, 370–376 (2020).

7. Zhao, T. *et al.* Facile synthesis of high tap density ZnO microspheres as advanced anode material for alkaline nickel-zinc rechargeable batteries. *Electrochimica Acta* **182**, 173–182 (2015).

8. Gan, W., Zhou, D., Zhao, J. & Zhou, L. Stable zinc anodes by in situ polymerization of conducting polymer to conformally coat zinc oxide particles. *J. Appl. Electrochem.* **45**, 913–919 (2015).

9. Wu, Y. *et al.* Ion-Sieving Carbon Nanoshells for Deeply Rechargeable Zn-Based Aqueous Batteries. *Adv. Energy Mater.* **8**, 1802470 (2018).

10. Zhang, Y. *et al.* Sealing ZnO nanorods for deeply rechargeable high-energy aqueous

battery anodes. *Nano Energy* **53**, 666–674 (2018).

11. Chen, P. *et al.* A deeply rechargeable zinc anode with pomegranate-inspired nanostructure for high-energy aqueous batteries. *J. Mater. Chem. A* **6**, 21933–21940 (2018).

12. Yan, Y. *et al.* A Lasagna-Inspired Nanoscale ZnO Anode Design for High-Energy Rechargeable Aqueous Batteries. *ACS Appl. Energy Mater.* **1**, 6345–6351 (2018).

13. Zhou, Z. *et al.* Graphene oxide-modified zinc anode for rechargeable aqueous batteries. *Chem. Eng. Sci.* **194**, 142–147 (2019).

14. Zhang, Y. *et al.* Deeply Rechargeable and Hydrogen-Evolution-Suppressing Zinc Anode in Alkaline Aqueous Electrolyte. *Nano Lett.* **20**, 4700–4707 (2020).
